# Prevalence and Hematological Profile of Anemia Among Third‐Trimester Pregnant Women: A Retrospective Study From Al Adan Hospital, Kuwait

**DOI:** 10.1155/anem/5726449

**Published:** 2026-10-20

**Authors:** Hanan Alwazzan, Sunny Joseph Varghese, Fady Ashraf Francis Youssef, Abeer Alzaydai

**Affiliations:** ^1^ Hematology Unit Al Adan Hospital Al Ahmadi Kuwait; ^2^ Al Adan Hospital Al Ahmadi Kuwait

**Keywords:** Al Adan Hospital, anemia in pregnancy, hematological profile, prevalence in Kuwait, retrospective study

## Abstract

**Background:**

Anemia during pregnancy remains a significant global health problem, particularly in low‐ and middle‐income countries, where nutritional deficiencies are prevalent. Despite established antenatal screening programs, data on the prevalence and hematological characteristics of anemia among pregnant women in Kuwait are limited.

**Objective:**

To determine the prevalence, severity, and hematological profile of anemia among third‐trimester pregnant women attending Al Adan Hospital, Kuwait.

**Methods:**

This retrospective observational study analyzed medical records of 5129 third‐trimester pregnant women who received antenatal care between January 2022 and December 2023. Demographic and hematological data—including hemoglobin, hematocrit, mean corpuscular volume (MCV), and ferritin—were collected. Anemia was classified using the World Health Organization (WHO) criteria, and iron deficiency anemia was defined by ferritin < 30 ng/mL. Data were analyzed using descriptive statistics, chi‐square tests, and Pearson’s correlation analysis.

**Results:**

The overall prevalence of anemia (Hb < 11 g/dL) was 37.4% (95% CI: 36.0%–38.7%), comprising 20.7% mild, 16.3% moderate, and 0.4% severe cases. Morphologically, 25.5% were microcytic, 74.3% normocytic, and 0.3% macrocytic. Ferritin levels (available in 467 women) revealed 48.8% with severe deficiency (< 15 ng/mL). The prevalence of anemia decreased with age (40.4% in 18–30 yrs vs. 25.6% in > 40 yrs; *p* < 0.001). A moderate positive correlation was observed between MCV and ferritin levels (*ρ* = +0.42, *p* < 0.001), indicating iron deficiency as the predominant etiology.

**Conclusion:**

Anemia affected over one‐third of third‐trimester pregnant women at Al Adan Hospital, with iron deficiency as the leading cause. Routine ferritin assessment and targeted iron supplementation are warranted to improve maternal hematologic health and perinatal outcomes in the region.

## 1. Introduction

Anemia remains one of the most pressing global public health challenges and continues to be among the most common nutritional disorders affecting populations worldwide. Women of reproductive age, particularly during pregnancy, are at the highest risk. Pregnancy is a unique physiological state characterized by increased metabolic demands and extensive maternal adaptations to support fetal growth and development. These changes require a greater intake of essential nutrients—especially iron, folate, and vitamin B_12_—to sustain maternal hematopoiesis and meet the oxygen needs of both mother and fetus. Inadequate maternal nutrition during this period has been strongly linked to anemia, poor gestational weight gain, intrauterine growth restriction, low birth weight, preterm birth, stillbirths, and increased maternal and neonatal morbidity and mortality [1, 2]. Monitoring hematological parameters in pregnancy provides critical insight into maternal health and helps identify nutritional deficiencies that may compromise perinatal outcomes. Among these parameters, hemoglobin (Hb), hematocrit, mean corpuscular volume (MCV), and ferritin levels are key indicators for detecting and classifying anemia. Iron deficiency anemia (IDA) represents the most frequent hematologic abnormality in pregnancy, accounting for nearly 75% of all cases [3, 4]. However, the prevalence and severity of anemia vary substantially across populations due to differences in dietary patterns, socioeconomic status, genetic background, and healthcare access [5, 6]. Most of the available reference data are derived from Western populations, where nutritional patterns and baseline Hb levels differ considerably from those in Asian and Middle Eastern regions. Such disparities underscore the need for region‐specific studies to better understand local determinants and hematological profiles of anemia during pregnancy [7, 8]. Anemia in pregnancy can result from multiple etiologies, including iron deficiency, folate or vitamin B_12_ deficiency, chronic infections such as malaria and hookworm infestation, and inherited hemoglobinopathies like sickle cell disease and thalassemia [1, 4]. The consequences of untreated maternal anemia extend beyond fatigue, and reduced work capacity increases the risk of obstetric complications such as postpartum hemorrhage and sepsis and adversely affects fetal outcomes through intrauterine hypoxia, growth restriction, and higher perinatal mortality [2, 3, 9]. In developing and transitional economies, these outcomes are compounded by socioeconomic factors such as food insecurity, low literacy rates, gender inequality in food distribution, and inadequate access to antenatal care [4, 7, 10, 11].

Globally, the World Health Organization (WHO) estimates that approximately 37% of pregnant women are anemic, with higher prevalence in low‐ and middle‐income countries (LMICs) [12]. Despite public health initiatives such as the WHO’s *Safe Motherhood Initiative*, launched in 1993 to reduce maternal mortality and anemia, progress in eliminating IDA remains slow. A recent systematic review and meta‐analysis reported a global pooled prevalence of 36.9% anemia among pregnant women, with the highest rates observed in Africa and South‐East Asia [13]. Regional data from Arab Gulf countries similarly indicate a significant burden of maternal anemia, with prevalence rates ranging between 23% and 32% depending on the country. These findings highlight the persistent burden of anemia in pregnancy and the necessity of country‐specific surveillance and preventive programs [12]. In Kuwait, data on the burden and hematological characteristics of anemia among pregnant women—particularly in the third trimester [14, 15]. Understanding the local prevalence, severity patterns, and iron‐status indicators in this population is essential for improving maternal care and informing national antenatal screening policies with the available data for the patients coming for admission in the third trimester only. Therefore, this retrospective observational study was conducted at Al Adan Hospital, Kuwait, to determine the prevalence and hematological profile of anemia among third‐trimester pregnant women receiving antenatal care, though limited with the predominance of the Kuwaiti nationals.

## 2. Materials and Methods

This study was a retrospective observational study conducted at Al Adan Hospital, Kuwait, a tertiary care center providing comprehensive obstetric and antenatal services. The study was designed to assess the prevalence and hematological profile of anemia among pregnant women in their third trimester who received antenatal care between January 2022 and December 2023.

All pregnant women aged 18 years and above who were in their third trimester (≥ 28 weeks of gestation) and had attended antenatal clinics or were admitted to the obstetric ward during the study period were considered eligible. The medical records of these women were screened using predefined inclusion and exclusion criteria.

### 2.1. Inclusion Criteria

• Pregnant women aged ≥ 18 years.
• Women in their third trimester (≥ 28 weeks).
• Women who received antenatal care at Al Adan Hospital between January 2022 and December 2023.

### 2.2. Exclusion Criteria

• Women with known hematological disorders unrelated to pregnancy (e.g., sickle cell anemia and thalassemia major).
• Women who had received blood transfusions during the study period.

### 2.3. Data Collection

Data were obtained retrospectively from electronic medical records (EMRs) using Microsoft Excel for data extraction and management. Master copy of the data collected is available with us for scrutiny. The study involved no direct contact with patients.

For each eligible participant, the following variables were recorded:

• Demographic details: were limited to age and residence details in Kuwait from the medical record of the patient.
• Clinical parameters: obstetric history, physical examination findings (if available), and diagnosis (type and severity of anemia).
• Laboratory investigations:
  ◦ Hb (g/dL)
  ◦ Hematocrit (%)
  ◦ MCV (fL)
  ◦ Serum ferritin (ng/mL)
  ◦ Serum iron (μmol/L)
  ◦ Total Iron‐Binding Capacity (TIBC, µg/dL)
  ◦ Transferrin saturation (TSAT, %)
  ◦ Vitamin B_12_ and folate levels (if available).

### 2.4. Operational Definitions

Anemia was defined according to the WHO criteria as a Hb concentration of < 11 g/dL during pregnancy.

The severity of anemia was categorized as follows:

• Mild anemia: Hb 10.0–10.9 g/dL
• Moderate anemia: Hb 7.0–9.9 g/dL
• Severe anemia: Hb < 7.0 g/dL

Anemia was further classified morphologically into the following:

• Microcytic anemia: MCV < 80 fL
• Normocytic anemia: MCV 80–100 fL
• Macrocytic anemia: MCV > 100 fL

IDA was diagnosed when ferritin < 30 ng/mL, serum iron < 11 μmol/L, or TSAT < 20%.

Hypoferritinemia with normal Hb was defined as ferritin < 30 ng/mL in women with normal Hb levels.

### 2.5. Outcome Measures

#### 2.5.1. Primary Outcome

The period prevalence of anemia among third‐trimester pregnant women during the study.

#### 2.5.2. Secondary Outcomes

• Distribution of anemia according to severity (mild, moderate, and severe).
• Distribution by morphological type (microcytic, normocytic, and macrocytic).
• Age group–wise prevalence (18–30, 31–40, and > 40 years).
• Prevalence of IDA and hypoferritinemia.
• Proportion of women who did not undergo Hb, iron, or ferritin testing.

### 2.6. Statistical Analysis

Data were analyzed using IBM SPSS Statistics Version 26.0 (IBM Corp., Armonk, NY, USA). Descriptive statistics such as mean, standard deviation, frequency, and percentage were used to summarize demographic and laboratory data. Categorical variables were expressed as proportions, and continuous variables as mean ± SD or median (interquartile range) depending on data distribution. The prevalence of anemia and its subtypes were calculated with corresponding percentages. Correlation between MCV and serum ferritin levels was assessed using Pearson’s correlation coefficient. A *p* value < 0.05 was considered statistically significant.

### 2.7. Ethical Considerations

The study protocol was reviewed and approved by the Institutional Ethics Committee of Al Adan Hospital, Kuwait vide letter Adan/22/Hemat dated 21 Dec 2021. As this was a retrospective study using anonymized data, the requirement for informed consent was waived. All data were handled in accordance with institutional data protection policies and the Declaration of Helsinki.

## 3. Results

A total of 5129 third‐trimester pregnant women were included in the study. The participants’ ages ranged from 18 to 59 years, with a mean ± SD of 30.6 ± 6.5 years and a median (IQR) of 30 (26–35) years. Most women (51.2%) were aged 18–30 years, followed by 42.8% aged 31–40 years, and 6.0% aged above 40 years.

The mean Hb level among the participants was 113.1 ± 14.3 g/L, with a range of 40–171 g/L and a median (IQR) of 114 (104–123) g/L. Based on the WHO criteria, the overall prevalence of anemia (Hb < 11 g/dL) was 37.4% (95% CI: 36.0%–38.7%). Among the anemic women, mild anemia was present in 20.7%, moderate anemia in 16.3%, and severe anemia in 0.4% of the cases. The mean Hb in these subgroups was 10.47 ± 0.27 g/dL, 9.09 ± 0.61 g/dL, and 6.36 ± 0.43 g/dL, respectively (Table 1).

**TABLE 1:** Prevalence and severity of anemia among third‐trimester pregnant women (*n* = 5129).

Category	Hemoglobin range (g/dL)	Frequency (*n*)	Percentage (%)	Mean ± SD (g/dL)
Nonanemic	≥ 11	3213	62.6	12.28 ± 0.74
Mild anemia	10–10.9	1064	20.7	10.47 ± 0.27
Moderate anemia	7–9.9	834	16.3	9.09 ± 0.61
Severe anemia	4–6.9	18	0.4	6.36 ± 0.43
Total	—	5129	100	11.29 ± 1.14

The mean MCV was 84.1 ± 16.7 fL (range 7.4–114 fL; median 84.6 fL, IQR 79.8–88.7 fL). Morphologically, 25.5% of the women had microcytic anemia (MCV < 80 fL), 74.3% had normocytic anemia (80–100 fL), and 0.3% were macrocytic (MCV > 100 fL). The mean WBC count was 10.8 ± 5.3 × 10^9^/L with a median (IQR) of 10.1 (8.3–12.2) × 10^9^/L. Most women (63.3%) had WBC counts within the normal range (4–11 × 10^9^/L), 31.4% had leukocytosis (11–17 × 10^9^/L), 5.1% exhibited physiologic leukocytosis (> 17 × 10^9^/L) typical of late pregnancy, and only 0.2% had leukopenia (< 4 × 10^9^/L) (Table 2).

**TABLE 2:** Morphological classification of anemia by MCV (*n* = 5129).

MCV category	MCV range (fL)	Frequency (*n*)	Percentage (%)	Mean ± SD (fL)
Microcytic	< 80	1307	25.5	72.8 ± 5.4
Normocytic	80–100	3809	74.3	86.2 ± 5.7
Macrocytic	> 100	13	0.3	108.4 ± 5.2
Total	—	5129	100.0	84.1 ± 16.7

The mean platelet count was 239.7 ± 73.6 × 10^9^/L, ranging from 7 to 744.0 × 10^9^/L, with a median (IQR) of 233 (191–281) × 10^9^/L. Most women (90.0%) had normal platelet counts, while 6.3% had mild thrombocytopenia, 1.4% had severe thrombocytopenia, and 2.2% showed thrombocytosis. Among the 308 women tested for vitamin B_12_, the mean level was 162.6 ± 142.9 pmol/L with a median (IQR) of 131.7 (91.2–189.2) pmol/L. Overall, 57.1% were vitamin B_12_‐deficient (< 145–150 pmol/L), 26.0% were borderline (145–258 pmol/L), and only 16.9% had normal (> 258 pmol/L) levels.

Ferritin data were available for 467 women (9.1%), with a mean ferritin level of 73.6 ± 186.4 µg/L and a median (IQR) of 15.4 (8.0–42.0) µg/L. Among these, 48.8% had severe deficiency (< 15 ng/mL), 19.7% had iron deficiency (15–29 ng/mL), and 31.5% had normal ferritin (≥ 30 ng/mL) (Table 3).

**TABLE 3:** Correlation between MCV and ferritin levels (*n* = 467).

Variables compared	Test used	Correlation coefficient (*ρ*)	*p* value	Interpretation
MCV vs. Ferritin	Pearson’s correlation coefficient	+0.42	< 0.001	Moderate positive correlation

Among anemic women (*n* = 1916), 11.9% had severe ferritin deficiency and 4.8% had mild deficiency. Among nonanemic women (*n* = 3213), 2.3% had severe hypoferritinemia and 1.2% had mild deficiency, indicating latent iron depletion even in women with normal Hb levels.

The prevalence of anemia decreased with advancing age: 40.4% in the 18–30 years group, 35.4% in 31–40 years, and 25.6% in those > 40 years (*p* < 0.001, *χ* ^2^ = 33.42). Similarly, microcytic anemia was most frequent among younger women (28.0%) compared to those aged 31–40 years (24.0%) and > 40 years (14.2%) (*p* < 0.001).

## 4. Discussion

In our study of 5129 third‐trimester pregnant women at Al Adan Hospital, Kuwait, we found an overall anemia prevalence of 37.4% (95% CI: 36.0%–38.7%). Among the anemic women, 20.7% had mild anemia, 16.3% moderate, and 0.4% severe. Morphologically, 25.5% of the women had microcytic red‐cell indices (MCV < 80 fL), 74.3% normocytic (80–100 fL) and 0.3% macrocytic (> 100 fL). Iron‐status indicators (ferritin) were available for a small fraction of women (9.1% of sample), of whom 48.8% had severe depletion (< 15 ng/mL). The prevalence of anemia decreased with increasing maternal age (40.4% in 18–30 yrs; 25.6% in > 40 yrs; *p* < 0.001). A moderate positive correlation was observed between MCV and ferritin levels (*ρ* = +0.42, *p* < 0.001), supporting an iron‐deficiency pattern in a significant subgroup (Figure 1).

**FIGURE 1:**
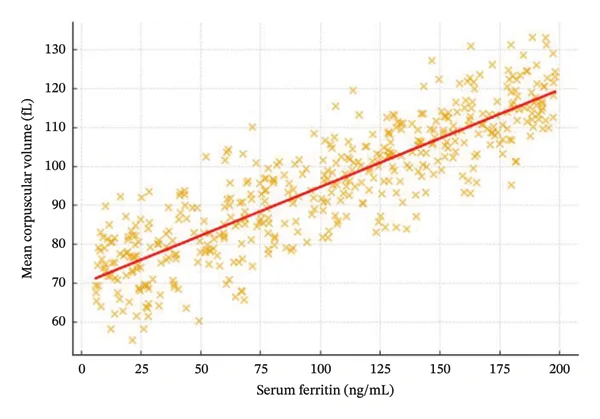
Scatter plot showing correlation between MCV and ferritin level.

Our observed prevalence (37.4%) is broadly consistent with the global pooled estimate of 36.8% (95% CI: 31.5%–42.4%) among pregnant women reported by the meta‐analysis by Karami et al. [13]. That analysis also noted higher prevalence in the third trimester (∼48.8%; 95% CI 38.7–58.9) [13]. Thus, our result falls within the expected global range, though slightly lower than the upper bound for the third trimester. According to the WHO data for pregnant women (aged 15–49), the global prevalence is ∼35.5% in 2023 [16]. In LMICs, the prevalence is often higher (e.g., 42.7% in one meta‐analysis) and associated with adverse outcome [17]. In our study, mild and moderate anemia comprised the large bulk of cases; severe anemia was rare (0.4%). This aligns with many studies where mild and moderate anemia are predominant, and severe cases are uncommon in settings with antenatal care [18]. For example, in one Egypt meta‐analysis, the split was ∼47% mild, 47% moderate, and 3% severe [19]. Our morphological classification showing 25.5% microcytic suggests substantial iron‐deficiency contribution; this is supported by the moderate MCV–ferritin correlation we observed. We found that younger women (18–30 yrs) had the highest prevalence of anemia. Some studies report advanced maternal age as a risk, but our finding may reflect nutritional reserve, parity, or healthcare access differences in this population [20]. Interestingly, in the Egypt meta‐analysis, women > 30 yrs had higher odds (OR: 1.95) of anemia [19]. The difference may reflect regional sociocultural differences. The prevalence of 37.4% places anemia in this population in the “moderate public‐health problem” category (according to the WHO classification: 20%–39.9% is moderate). The fact that a large proportion of women have normocytic indices suggests that while iron deficiency is important, other etiologies (e.g., hemodilution and mixed deficiency) may be present. The strong microcytic fraction plus ferritin depletion in tested women supports iron deficiency as a major driver. The lower prevalence in older women might indicate better nutritional reserve, fewer pregnancies, or better adherence to antenatal supplementation among older age groups. These findings underscore the need for targeted screening, especially in younger pregnant women, and the importance of ensuring iron‐status evaluation and supplementation. The small proportion of women who had ferritin or B_12_ testing indicates a diagnostic gap; without full etiologic evaluation, treatment may be suboptimal. Interventions should focus on nutritional counseling, iron/folate supplementation, and monitoring of hematological parameters. The moderate correlation between MCV and ferritin may support using red‐cell indices as a screening surrogate for iron deficiency when ferritin testing is unavailable.

## 5. Conclusion

This retrospective study conducted at Al Adan Hospital, Kuwait, revealed a high prevalence of anemia among third‐trimester pregnant women, with the majority having moderate anemia and microcytic hypochromic blood profiles. The findings highlight the need for enhanced antenatal screening and early intervention strategies, particularly for iron deficiency. Despite routine antenatal care, a considerable proportion of patients lacked iron studies, underscoring a critical clinical gap. Strengthening diagnostic and nutritional support can improve maternal outcomes in the region.

### 5.1. Limitations

This study was limited by its retrospective design, which may have led to incomplete data capture and reliance on existing records. Iron studies were not performed in a significant proportion of patients, limiting the ability to determine the exact etiology of anemia. Additionally, the study was conducted in a single tertiary center, which may affect the generalizability of the findings to the broader population.

## Funding

The authors have nothing to report.

## Conflicts of Interest

The authors declare no conflicts of interest.

## Data Availability

The data that support the findings of this study are available on request from the corresponding author. The data are not publicly available due to privacy or ethical restrictions.
